# Acupoint Catgut Embedding for Obesity: Systematic Review and Meta-Analysis

**DOI:** 10.1155/2015/401914

**Published:** 2015-08-31

**Authors:** Taipin Guo, Yulan Ren, Jun Kou, Jing Shi, Sun Tianxiao, Fanrong Liang

**Affiliations:** ^1^School of Acupuncture-Moxibustion and Tuina and Rehabilitation, Yunnan University of Traditional Chinese Medicine, Kunming 650500, China; ^2^School of Acupuncture-Moxibustion and Tuina, Chengdu University of Traditional Chinese Medicine, Chengdu 610075, China; ^3^Yunnan Province Hospital of Traditional Chinese Medicine, Kunming 650021, China

## Abstract

Acupoint catgut embedding (ACE) was applied widely to antiweight in China. The aim of this review is to estimate the effectiveness and safety of ACE on obesity. A literature search was conducted in PubMed, Cochrane Library, EBASE, CNKI, and so forth, using combination subject terms of obesity (or overweight, weight loss, etc.) and acupoint catgut embedding (or catgut implantation, catgut embedding). Improvement rate, reduction of body weight and body mass index (BMI), and so forth were analyzed. 43 studies were included for systematic review and meta-analysis. Although with poor methodological quality, ACE was superior to manual acupuncture (MA), sham, and cupping in improvement rate and presented a better tendency (OR > 1) compared with drugs and electroacupuncture (EA). Mean values of weight loss by ACE were 1.14 kg, 1.26 kg, 1.79 kg, and 3.01 kg comparing with MA, drugs, EA, and sham, respectively. Mean of BMI reduced to 0.56 kg/m^2^, 0.83 kg/m^2^, 0.79 kg/m^2^, and 1.63 kg/m^2^ comparing with MA, drugs, EA, and sham. Less adverse effects were reported. Pooled outcomes presented a tendency of equal or superior effects to other interventions and fewer side effects. Future high quality trials with rigorous design and positive FDA approved drug as control are urgent to assess the effect of ACE for obesity. PROSPERO registration number is as follows: CRD42015016006.

## 1. Introduction

Obesity, a common kind of metabolic disease, is characterized by redundant accumulation and abnormal distribution of fat. With transformation of modern lifestyle and diet structure, such as more intake of refined food and less physical activity, the prevalence of overweight and obesity is increasing amazingly in either developed countries or developing ones. Particularly in the last decade, the growth rate of obesity has ascended exponentially. For example [1, 2], the morbidity was 10%~40% in most European countries, and it was up to 35.5% in 2009~2010 in America in contrast to 30.5% in 1999~2002 and 22.9% in 1988~1994. With the world's most populous country being China, the obesity morbidity was just 1.5% in 1992, 7.1% in 2002, and up to 18% in 2011 [3, 4], and in some region it reached 37.71% [5]. According to the prediction [6] of World Health Organization (WHO), 2.3 billion people may suffer overweight and 0.7 billion get obesity.

According to WHO report in 2005 [6], obesity was deemed one of the top ten risk factors for many diseases like hypertension, diabetes, cardiovascular disease, stroke, and many cancers, and nearly 2.6 million people died directly due to obesity or overweight every year. Studies [7, 8] by working group on obesity in China (WGOC) revealed the morbidity rate of hypertension was 2.5 times higher when the body mass index (BMI) ≥ 24 kg/m^2^ than when BMI < 24 kg/m^2^ and 3.3 times higher with BMI ≥ 28 kg/m^2^ than with BMI < 24 kg/m^2^. Specifically, central obesity seemed more dangerous than systematic obesity, and even with mild obesity the morbidity and mortality of coronary heart disease increased when the waist circumference got bigger [9–11]. So, health problem is superior to aesthetics in obesity, and it urges finding a nice treatment.

Although the etiology and pathogenesis are still unclear, many clinical practice guidelines have been developed worldwide by relative medical and health organizations based on the existing evidences. For instance, with the American Clinical Guidelines of Overweight and Obesity released by National Heart, Lung and Blood Institute (NHLBI) of National Institutes of Health (NIH) since 2000 and with the reassessment of new evidences by American College of cardiology (ACC), American Heart Association (AHA), and NHLBI, a new version of guideline for the management of overweight and obesity in adults bas been made in 2013 [12]. Besides, Canada, China, and Europe have also published their prevention guides, which promoted the concern and management of obesity [4, 13, 14].

Obviously, the therapies of obesity are much similar in all the guidelines, consisting of the lifestyle modification of diet and exercise, drug, surgery, and complication therapy. Restriction of high calorie diet intake and increase of physical activity are recognized as the primary and most valid type of antiobesity, particularly for children because of the prohibition of drug and surgery in children's weight loss by American Food and Drug Administration (FDA). Studies [15, 16] also showed reduction of higher energy food intake such as high glucose and high fat and/or increase of physical activity could improve bodily functions and reduce fat. However, Cochrane system review [17] indicated that, due to insufficient longer-term evidences, the short-term adjustment of food consumption and movement was difficult to achieve sustained weight reduction. The change of diet and activity habit shaped for many years was difficult to adhere to for a long time, and this led to the failure of weight loss for weight regain [18].

So far, American FDA approved only 4 short-time use drugs as phentermine, diethylpropion, phendimetrazine, and benzphetamine and 3 medium- and long-time use ones as orlistat, lorcaserin, and phentermine plus topiramate-ER. Although these antiweight drugs were tested to be effective by comparison with placebo, there are 5% of them that were invalid [19]. Besides, there were so many obvious side effects like headache, dizziness, nausea and vomiting, insomnia, dry mouth, taste alteration, diarrhea, constipation, hypoglycemia, and change of cognition that the harm brought about by them was more than obesity itself, and these drugs frequently failed in decrease of cardiovascular morbidity and mortality and medical costs in the long run [19]. The efficacy and safety were still under suspicion, and it may be related to the ambiguity of obesity pathogenesis that the drug action was hard to selectively cut down the adipose tissue and there was no harm of health at molecular level [20]. The operative treatment of obesity was intended for obese adult with serious complication specifically caused by the excess of adipose cell like metabolic syndrome, and the surgical sites were mostly restricted at stomach, duodenum, pancreas, and gallbladder to decrease or constrain the function of digestive system [21].

Considering the side reaction and that there is no benefit to cardiovascular risks in antiobesity drugs and the high risk and narrow use of surgery, more clinicians have applied complementary and alternative therapy including TCM to lose weight [22].

A study showed there were 1088 articles of weight loss using TCM in CNKI database by 2012, and most of the methods were herbs and acupuncture [23]. Chinese herb was used to strengthen spleen and *qi* and have bowel movement, and radix astragali, bighead* Atractylodes* rhizome, and rhubarb were the most used ones [24]. However, the side effects like lack of strength and anorexia were difficult to avoid [25].

RCTs [26–30] manifested acupuncture was useful to reduce BMI, waist, and abdomen circumference and improve the quality of life, featuring less side effects, multifarious intervention means like ACE, auricular needle, EA, hand acupuncture, auricular plaster therapy, and so on. A review [31] of RCTs indicated acupuncture seemed more effective comparing to western antiobesity drugs; the mean of weight reduction was 0.65 kg by acupuncture and 0.08 kg by TCM drugs, and the mean of BMI reduction was 0.83 kg/m^2^ by acupuncture and 0.18 kg/m^2^ by TCM drugs.

However, owing to the long-term adherence of antiweight drugs, the conflict of time between treatment and daily work, and high expense of treating, more patients abandoned therapy. Hence, the method of ACE, developed from TCM acupuncture with a certain section of absorbable catgut suture implanted in acupoint, characterized by easy operation, durable and strong stimulation, and long interval between each treatment, has broadly been used to lose weight in China. Despite lack of effectiveness evaluated and normative management plan, most Chinese TCM hospitals and weight loss institutions have conducted ACE to treat obesity based on their own experience. To estimate the safety and effect appeared to be especially important, and it was also necessary to provide a treatment suggestion based on current evidences. The primary aims of this systematic review are to estimate the effectiveness and safety of ACE on obesity and formulate a treatment suggestion.

## 2. Methods 

### 2.1. Study Selection (Inclusive and Exclusive Criteria)

#### 2.1.1. Types of Study

To evaluate the curative effects of ACE on obesity and weight loss, this review was confined to RCTs comparing ACE with a control group, which contained drug, no treatment, placebo, diet and exercise therapy, and other types of acupuncture like MA, EA, ear auricular pressure treatment, acupoint pressure, and so forth. It is deemed a randomized study if the trial stated the “randomization” phrase, and the blinding was not restricted. Besides, Chinese and English were the limitation of language. The animal mechanism studies, case reports, self-pre- and postcontrol, or non-RCTs were excluded.

#### 2.1.2. Types of Participants

It included the participants with no limitation of age, gender, and type of overweight or obesity, including children obesity and abdominal obesity. The definitions of obesity or overweight using BMI, body weight, or percentage of weight excess compared with ideal weight were included. Patients with severe medical conditions, who are pregnant, and with drug-induced obesity were excluded.

#### 2.1.3. Types of Intervention

Clinical trials estimating the treatment of ACE used alone were included. Studies with cointerventions of drugs and other types of acupuncture such as MA, EA, massage, pressure, and laser acupuncture were included if the same intervention as control and other cointerventions were excluded. The control interventions with other types of acupuncture, drugs, no treatment (wait-listed or treatment as usual), placebo (no catgut implanted), and diet or physical activity therapy were included. Studies to compare the effect of difference of catgut length, operation, or acupoint prescription were excluded.

#### 2.1.4. Types of Outcome Measures

The primary outcomes consisted of improvement rate, reduction of body weight, BMI, hip circumference (HC), and waist circumference (WC). Secondary outcomes included the side effects, such as bleeding, serious discomfort, subcutaneous nodules, and infection. Treatment suggestions including frequency of acupoint prescription, frequency of treatment time, and course were also shown according all the included RCTs.

### 2.2. Data Sources and Search Methods

A literature search was conducted up to November 2014 in the databases of PubMed, Cochrane Library, EBSCO, Web of Science, EBASE, Springer, WHO International Clinical Trials Registry Platform (ICTRP), CNKI, Wanfang, CBM, and VIP, using the combination subject terms of obesity (or overweight, weight loss, weight control, weight reduction, and slim) and acupoint catgut embedding (or catgut implantation, catgut embedding). The item of RCT was also chosen in corresponding databases and the languages of Chinese and English were restricted.

#### 2.2.1. Data Extraction and Quality Assessment

Each literature of title and abstract was scanned by two reviewers (Taipin Guo and Sun Tianxiao) who have been trained and gained certifications in Chinese Cochrane Centre. All relevant articles of full text were investigated. The extracted information included descriptions of studies, characteristics of participants, interventions of both observation group and control group, adverse effect, and quality. Risk of bias was used to evaluate the quality of study. The decision of risk was made by two reviewers. If inconsistent results appeared, the final decisions were made by all the authors. For missing or ambiguous data, we tried to contact the author as possible, and for duplicate publication we only selected the original.

#### 2.2.2. Measures of Publication Bias and Treatment Effect

Review Manager (version 5.1, the Nordic Cochrane Centre, Copenhagen, Denmark) was applied to assess curative effect and publication bias. Forest plot was used to illustrate the relative strength of curative effect. Meanwhile, according to Cochrane handbook suggestion, the funnel plot was pictured to describe publication bias visually as the number of trials was more than 10. There was no publication bias as a symmetric inverted funnel while the publication bias or a systematic difference of small or big sample size effects existed as an asymmetric funnel. The heterogeneity result was indicated using *I* ^2^ values, and random effect model was chosen when *I* ^2^ > 50% or fixed effect model when *I* ^2^ < 50%. An odds ratio (OR) > 1 suggested greater reduction of body weight (≥2 kg) or BMI (≥0.5 kg m^−2^) in the ACE group than control group in calculation of discrete data. The calculation of mean differences of changes in body weight and BMI between ACE and control groups was also conducted. Because of most literatures showing only pre- and posttreatment values, mean change was obtained by subtracting pretreatment from posttreatment values and standard deviation (SD) change was calculated by the given pre- and posttreatment SD according to Cho's formula [75].

## 3. Results

### 3.1. Study Description and Participants

**Figure 1:**
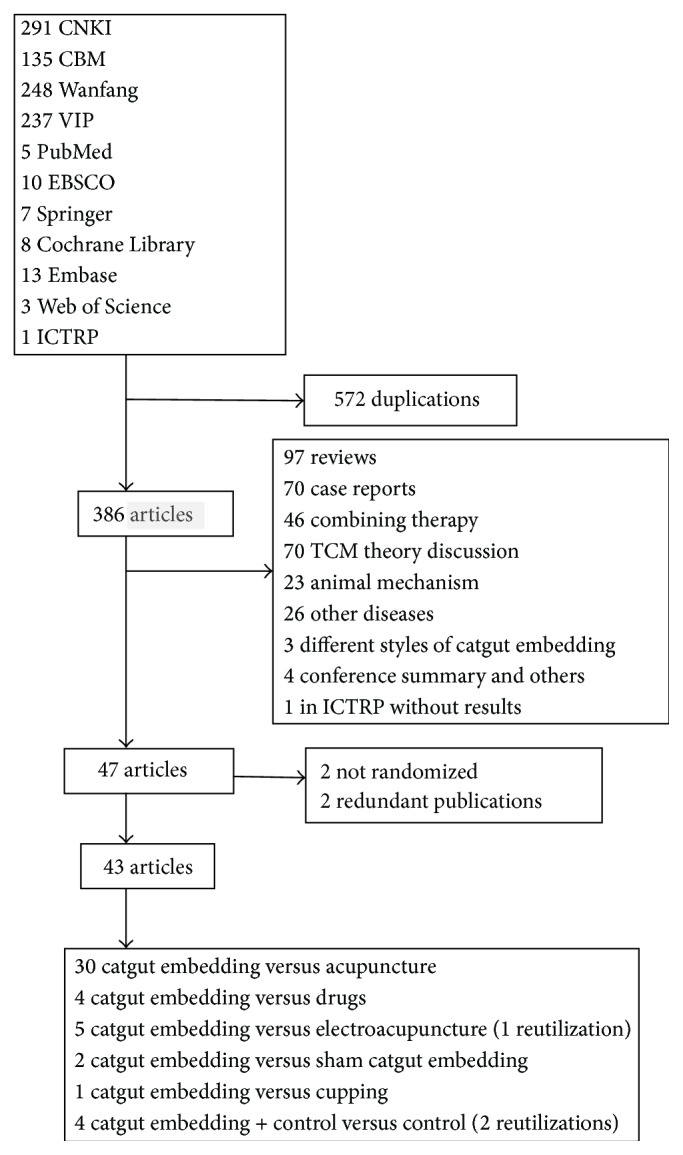
Flow diagram or the number of studies included and excluded.

Our initial search identified 958 probable articles from the databases, of which 386 were reserved with 572 excluded for duplication. 47 articles were selected at the scan of titles and abstracts based on the inclusive and exclusive criteria. Finally, 43 studies with 3520 participants met the inclusion criteria and were included to this systematic review with 2 nonrandomizations and 2 redundant publications eliminated by full text view. In these 43 trials, there were 30 articles [32–61] reporting the weight loss effect of ACE (1241 patients) with MA (1096 patients), 4 ACE (153 patients) versus drugs (165 patients) [62–65], 5 ACE (155 patients) versus EA (155 patients) [58, 66–69], 2 ACE (88 patients) versus sham (88 patients) (that with the same operation as ACE but the catgut was not implanted [70, 71]), 1 ACE (40 patients) versus cupping (40 patients) [72], 2 ACE plus EA (66 patients) versus EA (57 patients) [73, 74], and 2 ACE plus MA (91 patients) versus MA (85 patients) [42, 43], and all the included trials were from China. The articles were filtrated as shown in Figure 1.

### 3.2. Risk of Bias in Included Studies

**Figure 2:**
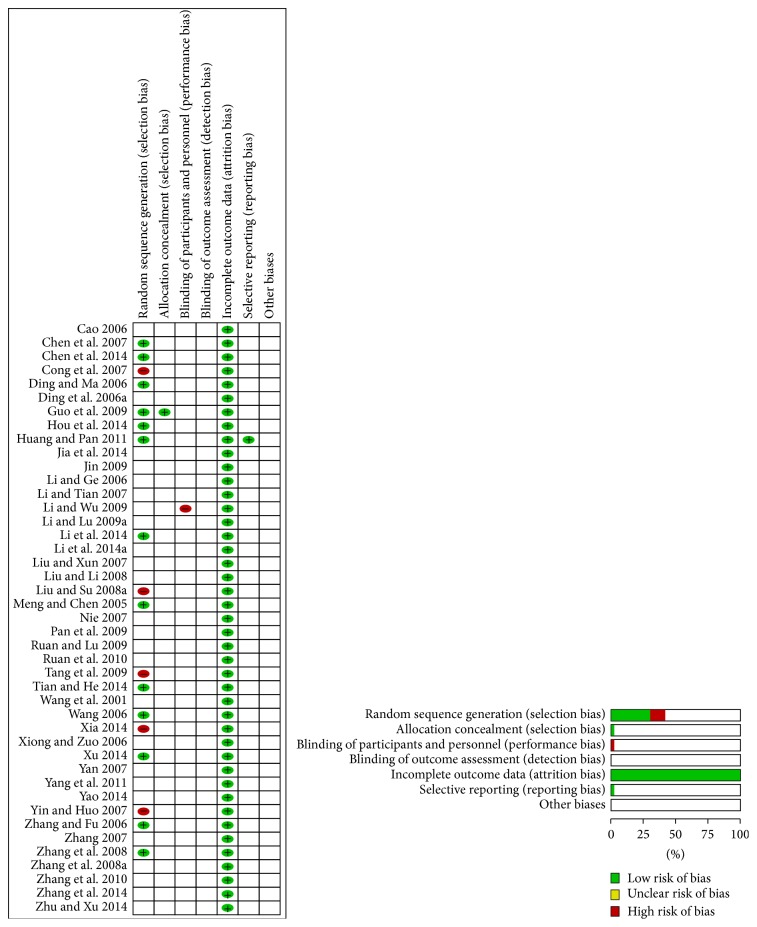
Risk of bias summary and graph.

As shown in Figure 2, the methodological quality of all the 43 articles was poor and probably in high risk with almost no reports of both allocation concealment and blinding of participants, acupuncturists, or statisticians except 1 reported, respectively [38, 70]. Only 18 of 43 reported the random sequence generation with 13 [33–35, 41, 46, 48, 50, 53, 60, 61, 68, 70, 72] in low risk and 5 [44, 51, 57, 63, 74] in high risk. The simple size varied from 20 to 150 participants (20 to 150 participants in ACE groups and 20 to 90 in control groups). Two articles [63, 70] reported a small proportion of dropout whose data was also excluded from analysis, but the reasons were not given or clearly described. None of them stated the calculation of sample size. More details were reported in Table 1 based on EBM PICOs (patient, intervention, control, and outcomes) principle. Because all of the studies did not publish the trial protocols or registration, the selective reporting of outcomes cannot be judged.

### 3.3. Comparison 1: ACE versus MA

#### 3.3.1. Frequency of Improvement

**Figure 3:**
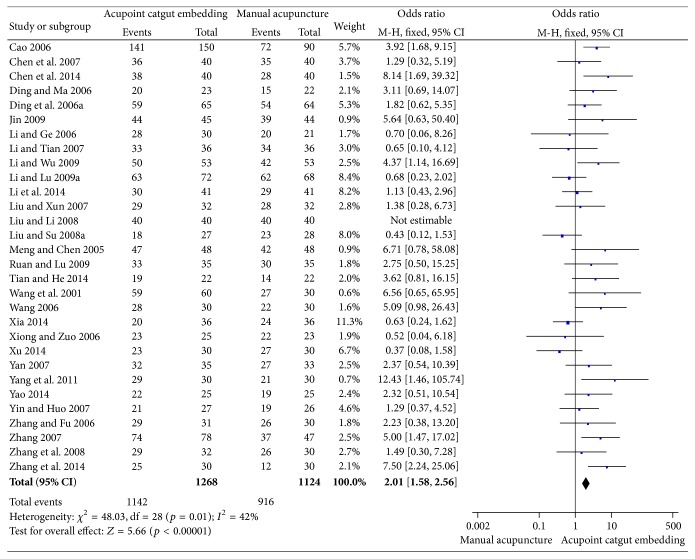
Forest figure of the frequency of improvement in the comparison of ACE versus MA.

**Figure 4:**
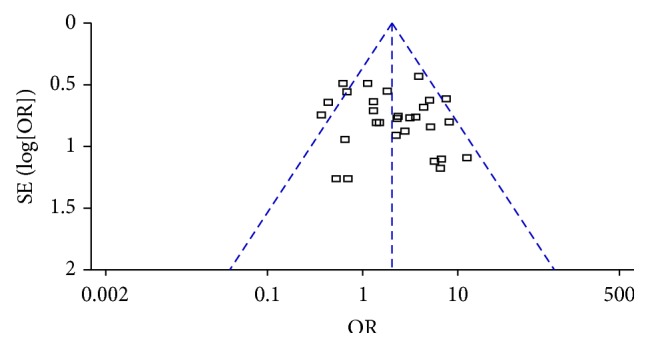
Funnel plots of the frequency of improvement in the comparison of ACE versus MA.

There were 30 trials with 2392 patients [32–61] in the comparison of ACE versus MA and all of them evaluated the frequency of improvement. The heterogeneity within each trial was low (*I* ^2^ = 42%, Chi^2^ test *p* = 0.01), and fixed effect model was applied to calculate the incorporated data. The pooled outcomes showed more improvement of obesity participants in ACE groups than in MA groups (OR = 2.01, 95% CI = 1.58~2.56, *p* < 0.01) (Figure 3). The symmetry was shown in funnel plot and indicated low publication bias (Figure 4).

#### 3.3.2. Reduction of BMI and Body Weight

**Figure 5:**
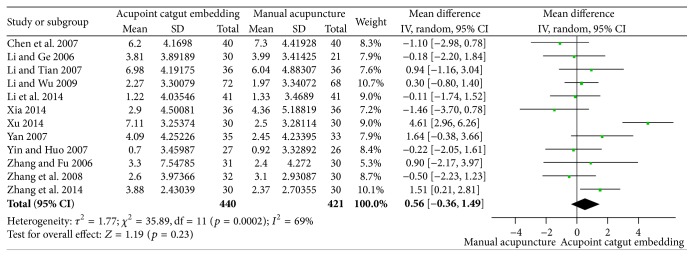
Forest figure of BMI loss in the comparison of ACE versus MA.

**Figure 6:**
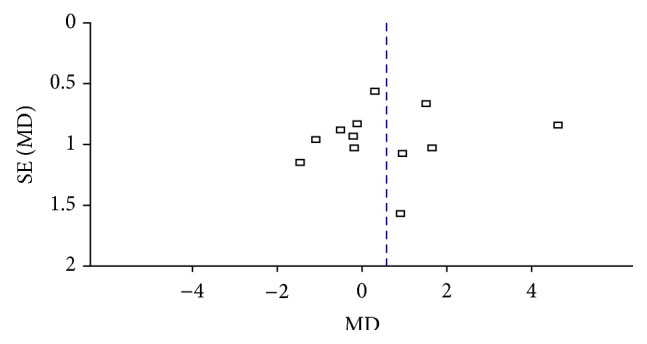
Funnel plots of BMI loss in the comparison of ACE versus MA.

12 studies reported the decline of BMI [33, 39–42, 51, 53, 54, 57, 58, 60, 61], and no difference was found between the two groups of catgut embedding versus MA (MD = 0.56, 95% CI = −0.36~1.49, *p* = 0.23) tested by random effect model for their statistic heterogeneity (*I* ^2^ = 69%, Chi^2^ test *p* = 0.0002) which may be caused by the differences of frequency of intervention, manipulations, and participants (Figure 5). Publication bias was presented optically by the asymmetry of funnel plot (Figure 6).

**Figure 7:**
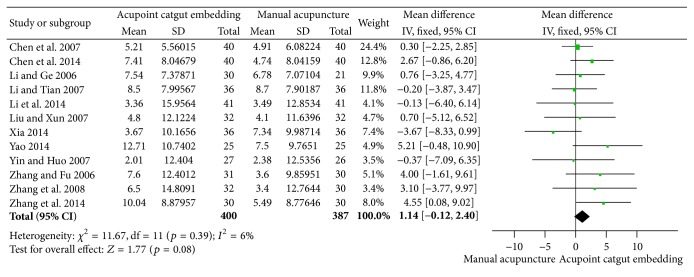
Forest figure of body weight loss in the comparison of ACE versus MA.

**Figure 8:**
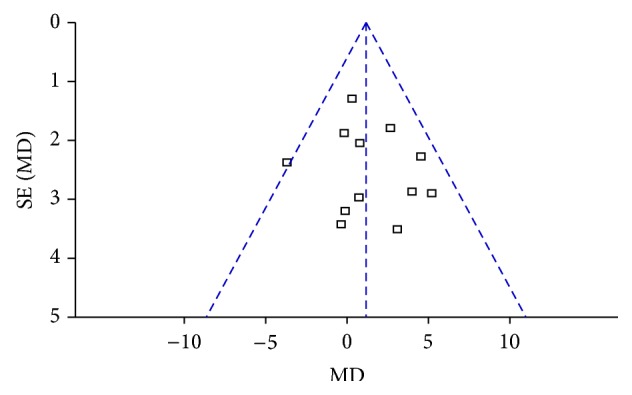
Funnel plots of body weight loss in the comparison of ACE versus MA.

For the reduction of body weight, as shown in Figure 7, the merged results of 12 studies [33, 34, 39–41, 45, 51, 56–58, 60, 61] demonstrated no variance in the two groups using a fixed effects model (MD = 1.14, 95% CI = −0.12~2.40, *p* = 0.08). No heterogeneity (*I* ^2^ = 6%, Chi^2^ test *p* = 0.39) and their publication bias were found in Figure 8.

#### 3.3.3. Reduction of WC and HC

**Figure 9:**
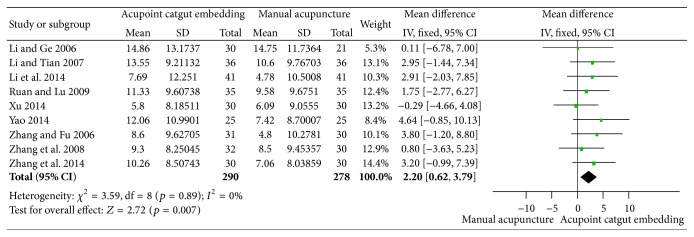
Forest figure of WC loss in the comparison of ACE versus MA.

**Figure 10:**
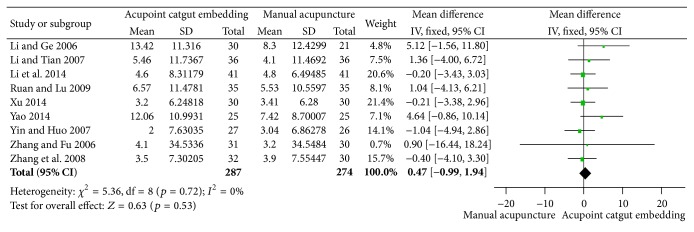
Forest figure of HC loss in the comparison of ACE versus MA.

The combined reduction of WC from 9 trials [39–41, 47, 53, 56, 58, 60, 61] was of significant difference between the two groups (MD = 2.20, 95% CI = 0.62~3.79, *p* = 0.007), and no significant heterogeneity was found (*I* ^2^ = 0%, Chi^2^ test *p* = 0.89), as shown in Figure 9. However, the therapy of ACE was not superior to MA according to the pooled outcome of HC (MD = 0.47, 95% CI = 0.99~1.94, *p* = 0.53); no significant heterogeneity (*I* ^2^ = 0%, Chi^2^ test *p* = 0.72) was shown in Figure 10.

### 3.4. Comparison 2: ACE versus Drug

#### 3.4.1. Frequency of Improvement

**Figure 11:**
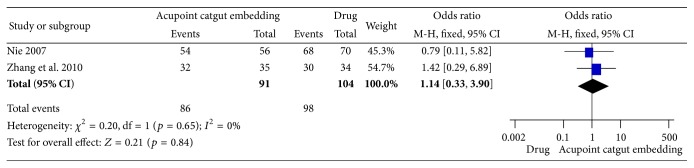
Forest figure of frequency of improvement in the comparison of ACE versus drug.

**Figure 12:**
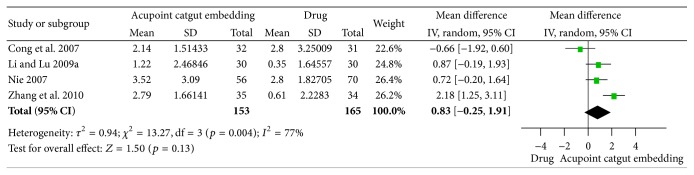
Forest figure of BMI loss in the comparison of ACE versus drug.

Two trials [62, 64] reported the frequency of improvement and there was no difference between ACE group and drug group (OR = 1.14, 95% CI = 0.33~3.90, *p* = 0.84). No significant heterogeneity was tested among the results (*I* ^2^ = 0%, Chi^2^ test *p* = 0.65) (Figure 11).

(*1) Reduction of BMI and Body Weight*. Four trials [62–65] reported the reduction of BMI, and the combined results indicated no significant difference between the two interventions (OR = 0.83, 95% CI = −0.25~1.91, *p* = 0.84). It was considerably heterogeneous among the 4 studies (*I* ^2^ = 77%, Chi^2^ test *p* = 0.004) and might be caused by the difference in drugs or frequency of ACE (Figure 12).

**Figure 13:**
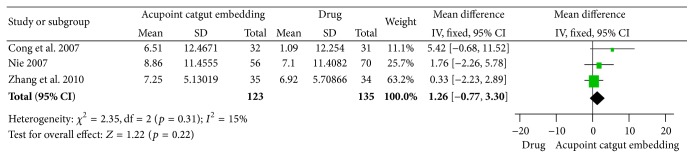
Forest figure of body weight loss in the comparison of ACE versus drug.

The pooled results of 3 studies [62–64] showed that there was no significant difference about body weight loss between the intervention of ACE and drugs (MD = 1.26, 95% CI = −0.77~3.30, *p* = 0.22). There was no significant heterogeneity between the studies (*I* ^2^ = 15%, Chi^2^ test *p* = 0.31) (Figure 13).

**Figure 14:**
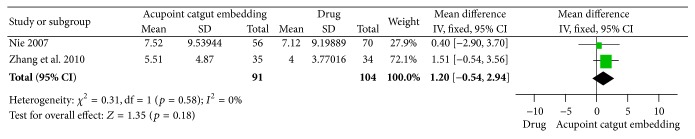
Forest figure of WC loss in the comparison of ACE versus drug.

**Figure 15:**
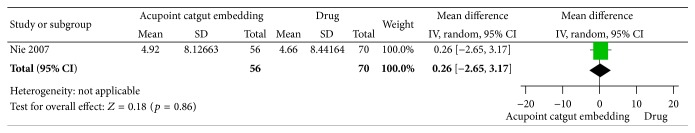
Forest figure of HC loss in the comparison of ACE versus drug.

*(2) Reduction of WC and HC.* For the outcome of WC reduction, there were 2 trials [62, 64] reported and no significant difference by their combination (MD = 1.20, 95% CI = −0.54~2.94, *p* = 0.18). There was no heterogeneity between the results (*I* ^2^ = 0%, Chi^2^ test *p* = 0.58) (Figure 14). One trial [62] reported the decrease of HC, and no difference between the two groups was shown (MD = 0.26, 95% CI = −2.65~3.17, *p* = 0.86) (Figure 15).

### 3.5. Comparison 3: ACE versus EA

#### 3.5.1. Frequency of Improvement

**Figure 16:**
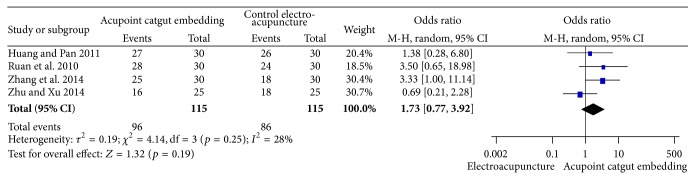
Forest figure of frequency of improvement in the comparison of ACE versus EA.

There was no statistical difference in frequency of improvement according to the combined results of 4 studies [58, 67–69] comparing the ACE with EA (OR = 1.73, 95% CI = 0.77~3.92, *p* = 0.19). Significant heterogeneity was not tested among the results (*I* ^2^ = 28%, Chi^2^ test *p* = 0.25) (Figure 16).

#### 3.5.2. Reduction of BMI and Body Weight

**Figure 17:**
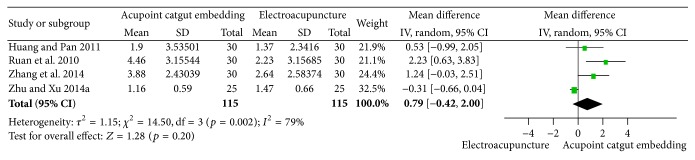
Forest figure of BMI loss in the comparison of ACE versus EA.

The pooled results of 4 trials [58, 67–69] released the idea that ACE treatment was not better than EA statistically in reduction of BMI (MD = 0.79, 95% CI = −0.42~2.00, *p* = 0.20). Substantial heterogeneity between the results was shown (*I* ^2^ = 79%, Chi^2^ test *p* = 0.002) and maybe explained the difference of patients or acupoint prescriptions (Figure 17).

**Figure 18:**
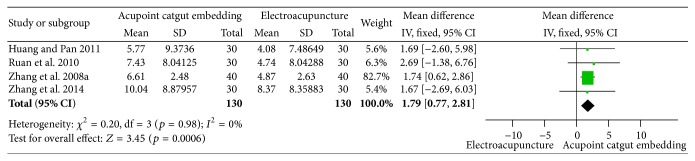
Forest figure of body weight loss in the comparison of ACE versus EA.

Significant difference of body weight loss (MD = 1.79, 95% CI = 0.777~2.81, *p* = 0.0006) was tested by the pooled results of 4 trials [58, 66, 68, 69]. There was no obvious heterogeneity among the results (*I* ^2^ = 0%, Chi^2^ test *p* = 0.98) (Figure 18).

#### 3.5.3. Reduction of WC and HC

**Figure 19:**
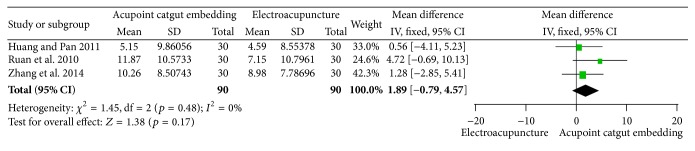
Forest figure of WC loss in the comparison of ACE versus EA.

**Figure 20:**
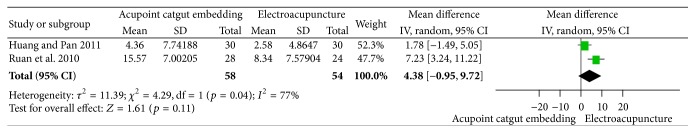
Forest figure of HC loss in the comparison of ACE versus EA.

Three studies [58, 68, 69] reported no difference in WC loss between the two interventions (MD = 1.89, 95% CI = −0.79~4.57, *p* = 0.17), and no heterogeneity was observed (*I* ^2^ = 0%, Chi^2^ test *p* = 0.49) (Figure 19). Two studies [68, 69] indicated there was no difference in HC loss between the two interventions (MD = 4.38, 95% CI = −0.95~4.72, *p* = 0.011), and heterogeneity was shown, maybe caused by differences of patients or acupoint prescriptions (Figure 20).

### 3.6. Comparison 4: ACE versus Sham

#### 3.6.1. Frequency of Improvement

**Figure 21:**
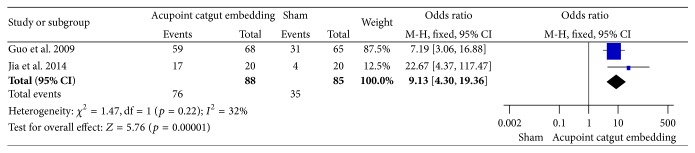
Forest figure of frequency of improvement in the comparison of ACE versus sham.

The pooled results of 2 trials [70, 71] showed there were significant differences (OR = 9.13, 95% CI = 4.30~11.36, *p* < 0.00001) in the improvement rate comparing ACE with sham in which the needling instrument was just penetrated but the catgut was not implanted. There was no heterogeneity among the results (*I* ^2^ = 32%, Chi^2^ test *p* = 0.22) (Figure 21).

#### 3.6.2. Reduction of BMI and Body Weight

**Figure 22:**
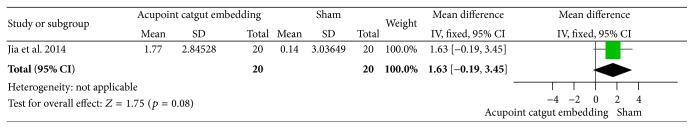
Forest figure of BMI loss in the comparison of ACE versus sham.

**Figure 23:**
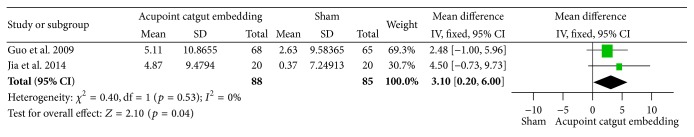
Forest figure of body weight loss in the comparison of ACE versus sham.

One study [71] reported the BMI loss, and no significant effect was observed (MD = 1.63, 95% CI = −0.19~3.45, *p* < 0.08) (Figure 22). The result of body weight loss was of significant difference (MD = 3.10, 95% CI = 0.20~6.00, *p* < 0.04) between the two interventions tested by merging the 2 studies [70, 71], and no heterogeneity among the results (*I* ^2^ = 0%, Chi^2^ test *p* = 0.53) (Figure 23) was tested.

#### 3.6.3. Reduction of WC and HC

**Figure 24:**
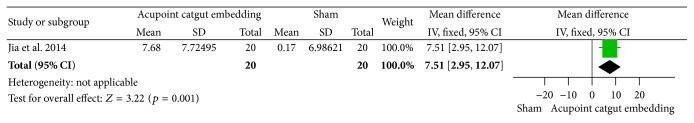
Forest figure of WC loss in the comparison of ACE versus sham.

**Figure 25:**
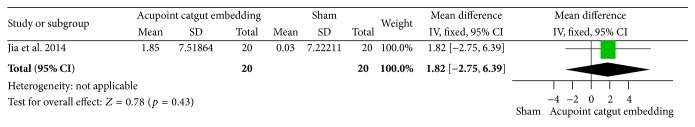
Forest figure of HC loss in the comparison of ACE versus sham.

Just 1 study [71] reported the loss of WC and HC, and the loss of WC was of significant difference (MD = 7.51, 95% CI = 2.95~12.07, *p* = 0.001) (Figure 24), while the loss of HC was not (MD = 1.92, 95% CI = −2.75~6.39, *p* = 0.43) (Figure 25) in the comparison of the two interventions.

### 3.7. Comparison 5: ACE versus Cupping Therapy

**Figure 26:**
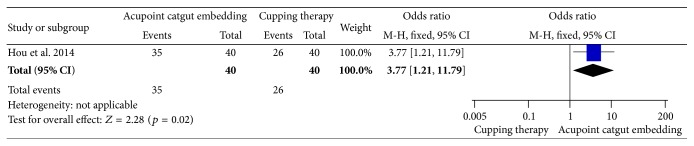
Forest figure of improvement rate in the comparison of ACE versus cupping therapy.

**Figure 27:**
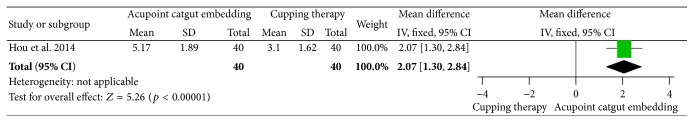
Forest figure of WC loss in the comparison of ACE versus cupping therapy.

Only 1 study reported the comparison of ACE with cupping therapy. ACE was superior to cupping therapy in improvement rate (OR = 3.77, 95% CI = 1.21~11.79, *p* = 0.02) (Figure 26) and WC loss (MD = 2.07, 95% CI = 1.30~2.84, *p* < 0.00001) (Figure 27), and other outcomes were not reported.

#### 3.7.1. Comparison 6: ACE Plus Control versus Control

We selected 4 combining therapy trials [42, 43, 73, 74] which could identify the ACE effect, and the improved rate of patient and the reduction of BMI and body weight were reported.

#### 3.7.2. Frequency of Improvement

**Figure 28:**
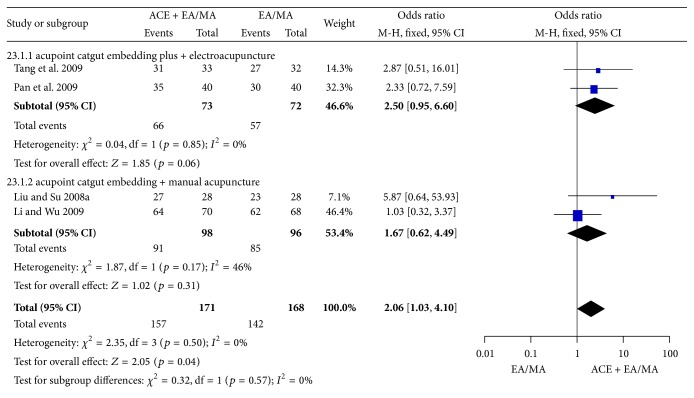
Forest figure of improvement rate in the comparison of ACE plus control versus control.

The effect rate was not different in both comparisons of ACE plus EA versus EA (OR = 2.50, 95% CI = 0.95~6.60, *p* = 0.06) from 2 trials [73, 74] with no heterogeneity (*I* ^2^ = 0%, Chi^2^ test *p* = 0.85) and ACE plus MA versus MA (OR = 1.67, 95% CI = 0.62~4.49, *p* = 0.31) from 2 trials [42, 43] with no heterogeneity (*I* ^2^ = 46%, Chi^2^ test *p* = 0.17). The significant improvement was shown when the two comparisons with 4 trials [42, 43, 73, 74] were combined (OR = 2.06, 95% CI = 1.03~4.10, *p* = 0.04), with no heterogeneity among results (*I* ^2^ = 0%, Chi^2^ test *p* = 0.50) (Figure 28).

#### 3.7.3. Reduction of BMI and Body Weight

**Figure 29:**
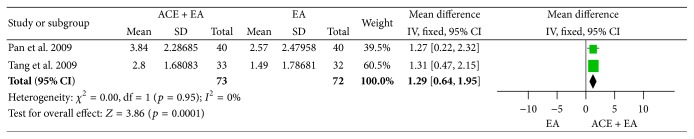
Forest figure of BMI loss in the comparison of ACE plus EA versus EA.

**Figure 30:**
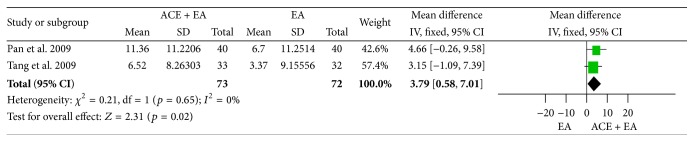
Forest figure of body weight loss in the comparison of ACE plus EA versus EA.

Two studies [73, 74] of comparisons between ACE plus EA and EA have both reported the changes of BMI and body weight. There were significant decreases of both BMI (MD = 1.29, 95% CI = 0.64~1.95, *p* = 0.0001) (Figure 29) with no heterogeneity (*I* ^2^ = 0%, Chi^2^ test *p* = 0.95) and body weight (MD = 3.79, 95% CI = 0.58~7.01, *p* = 0.02) with no heterogeneity (*I* ^2^ = 0%, Chi^2^ test *p* = 0.65) (Figure 30) in ACE plus EA group as opposed to EA group in line with the pooled results of the 2 trials.

### 3.8. Adverse Events

Among these studies, only 3 trials [62–64] reported the side effects in the comparison between ACE and drugs. Nie [62] reported headache, dry mouth, anorexia, insomnia, constipation, rapid heartbeat, and mild high blood pressure in sibutramine drug group. Cong et al. [63] reported subcutaneous indurations (*n* = 5), red and swollen (*n* = 1) in ACE group, and gastrointestinal discomfort (*n* = 6) in Chinese patent medicine ZhiBiTuo group. Zhang et al. [64] just reported 5 cases in sibutramine drug group that experienced side effects and no more details were reported.

### 3.9. Treatment Suggestion

**Figure 31:**
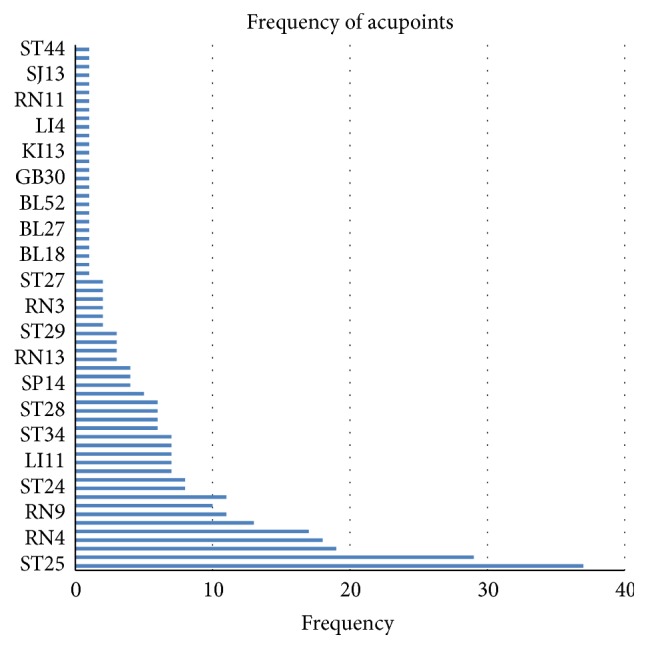
Frequency of acupoints in ACE.

A total of 63 acupoints have been extracted from all the included RCTs except for 2 trials [39, 42] of individual treatment for flexibility and 1 trial [35] not given, and the frequency of usage of them was listed as Figure 31. Obviously, the acupoints of ST25, RN12, ST40, RN4, RN6, SP15, RN9, SP6, and ST36 were the most used to lose weight. According to all the included RCTs, the frequency of treatment time ranged from 1 time per 35 days to 3 times per 1 week (alternate use to acupoints), and most of the frequencies were 1~2 time(s) per 7~15 days. The total times of treatment were varied from 2 to 24, and 4~8 times were most used. So, the treatment suggestion may be suitable as acupoints of ST25, RN12, ST40, RN4, RN6, SP15, RN9, SP6, and ST36, 1 time per week, lasting 4~8 times for 1~2 month(s).

## 4. Discussions

There were a total of 43 trials with 3520 patients included in this review, all of them from China. The therapy of ACE has been evaluated by comparing with sham, drugs, and other different intervention forms of acupuncture such as MA, EA, and cupping jar. Previous systematic reviews [31, 75] have excluded the trials of the contrasts of different types of acupuncture. In our opinion, it was also valuable to assess the effect of ACE comparing with other acupuncture interventions and a better choice may be provided for both doctors and obesity patients.

The overall quality of these identified trials was poor and in high risk of bias. Although all the trials have claimed randomization, most of them did not illustrate the generation of random sequence. It was hard to apply blinding of participants and acupuncturist. These trials seldom reported allocation concealment and blinding of participants, acupuncturist, and outcome assessment. The sample was also very small in most comparisons in this review.

Despite methodological quality and sample size limitations, all the pooled outcomes (improvement rate, loss of weight, BMI, WC, and HC) have presented a tendency of consistent superior effects of ACE or combined therapy comparing with other interventions (MA, EA, drugs, sham, and cupping), and less adverse effect was reported. There were different versions of evaluation standard to estimate the improvement rate, but all of them basically claimed the loss of body weight was more than 2 kg or BMI more than 0.5 kg/m^2^. Hence, the results could be combined. The pooled data of improvement rate displayed that ACE was more effective than MA, sham, and cupping (*p* < 0.05). The combined therapy of ACE with MA or EA was also better than MA or EA alone (*p* < 0.05) in improvement rate. Although in contrast with drugs or EA, the improvement rate was 3.79 kg compared with EA alone; forest figures visually described a better tendency in ACE group (OR > 1).

The mean reductions of body weight by ACE also might be more effective which were 1.14 kg, 1.26 kg, 1.79 kg, and 3.01 kg, respectively, contrasted to MA, drugs, EA (*p* < 0.05), and sham (*p* < 0.05). The mean reduction of body weight by ACE combined with EA was 3.79 kg than by EA alone (*p* < 0.05). Comparing with MA, drugs, EA, and sham, the mean BMI accordingly reduced to 0.56 kg/m^2^, 0.83 kg/m^2^, 0.79 kg/m^2^, and 1.63 kg/m^2^ in obesity treated by ACE. The BMI loss was 1.29 kg/m^2^ in ACE combined with EA group than EA alone (*p* < 0.05).

WC and HC were removed more by ACE comparing with control. WC reduction was generally supposed to be meaningfulness in prevention and treatment of diabetes and cardiovascular diseases [76]. HC was also regarded as important as WC in prediction of diabetes and cardiovascular diseases [77]. In this review, the mean decreases of WC were 2.20 cm, 1.20 cm, 1.89 cm, 7.51 cm, and 2.07, comparing with MA (*p* < 0.05), drugs, EA, sham (*p* < 0.05), and cupping (*p* < 0.05) correspondingly. Meanwhile, the changes of HC were 0.47 cm, 0.26 cm, 4.38 cm, and 1.82 cm comparing with MA, drugs, EA, and sham accordingly.

However, in the identified 4 trials [62–65] of comparison between ACE and drug, only 2 trials [62, 64] applied sibutramine as control which has been withdrawn from market by the American Food and Drug Administration (FDA) in 2010 for its potential cardiovascular risk [78], and the other 2 studies [63, 65] used metformin and Chinese patent medicine ZhiBiTuo for controls which did not belong to antiobesity drugs and had benefits of weight loss.

There was another review [79] that included 9 literatures from PubMed database which has reported the therapy of ACE using descriptive comments with a quantitative analysis and revealed the same result regarding its effect of equality or superiority comparing to control treatments. Furthermore, another 2 systematic reviews [31, 75] have reported results that acupuncture including the types of MA, EA, catgut embedding, and cotreatment was superior or similar to pharmacotherapy and diet control based on the pooled data, and the different effects among the different types of acupuncture are not included. Our results indicated that the effects of ACE were also greater than or equal to other kinds of acupuncture and other kinds of drug and nondrug therapy. In some sense, ACE might be more effective for obesity.

Lowering expense and time should be another advantage of ACE in the fast-paced society time. The treatment frequency of ACE is 1 time per week normally, while it is 3 times per week in MA or EA. Mostly, the patients will stay in clinical room more than 30 minutes every treatment time, but the therapy time should be less by ACE. According to Chinese cost of treating, it should be more economical by ACE than MA or EA, which has been proved by Huang and Pan [68].

In ancient TCM theory, obesity has been recorded since two thousand years in* Huangdi Neijing*, and the increasing intake of sweet and greasy foods is the main cause. The water and soil type of TCM constitutions are the susceptible population. The dysfunction of spleen and stomach is the essential reason. Obesity patient often suffers from the syndrome of *qi* deficiency and phlegm retardation [80, 81]. The therapeutic principles were tonifying *qi* and dissolving sputum, and the needle should penetrated deeply the acupoint and retained for a long time [81].

In this review, we have also sequenced the usage frequency of acupoint, and the acupoints of ST25, RN12, ST40, RN4, RN6, SP15, RN9, SP6, and ST36 were the most used. Distribution in abdomen and thigh hypertrophic muscles of spleen, stomach, and ren meridians is the characteristic of antiobesity acupoint prescription, which has the ability of strengthening spleen and eliminating dampness in TCM theory.

The limitations of this review were inferior quality of included trials and small sample size. Besides, most trials focused on the comparison of different types of acupuncture and reported less the positive drug as control. This limited the universality of the findings and acceptability. However, this review was the first of quantitative analysis of ACE, and the treatment advice was given.

## 5. Conclusions

Our review found the evidences that the effects of obesity treated by ACE were superior or equal to other interventions (MA, EA, drugs, sham, and cupping) based on the assessment of the pooled outcomes (improvement rate, loss of weight, BMI, WC, and HC). Further high quality studies with the rigorous designed and positive FDA approved drug as control are urgent to evaluate the effect of ACE for treating obesity.

## Figures and Tables

**Table 1:** Characteristics of RCTs of ACE for obesity.

First author, year	Intervention					Outcome (ACE versus Ct.)		Side effects	
	No. in ACE/Ct.	Acupoints of ACE	Types of Ct.	Frequency of ACE (total times)	Frequency of Ct. (total times)	With benefits	No effect	ACE	Ct.
Cao 2006 [32]	150/90	LI11, RN12, ST25, RN6, SP9, SP6, AShi	MA	1 per 20 days (3)	1 per 1-2 day(s) (45)	IR, BW, BMI	/	NR	NR

Chen 2007 [33]	40/40	ST34, RN12, ST25, RN9, ST40	MA	1 per 1 week (4)	1 per 1-2 day(s) (19)	/	/	NR	NR

Chen 2014 [34]	40/40	SI4, BL22, BL17	MA	1 per 1 week (2)	5 per 1 week (10)	IR, BW, FP	/	NR	NR

Ding 2006 [35]	23/22	Not Given	MA	1 per 10 days (3)	1 per 2 days (15)	IR	/	NR	NR

Ding 2006 [36]	65/64	RN12, ST25, ST40, LI 11	MA	1 per 10 days (3)	1 per 2 days (15)	IR	/	NR	NR

Jin 2009 [37]	45/44	RN12, RN10, RN6, RN5, RN4, SP15, ST25, ST26, ST24	MA	1 per 1 week (4)	1 per 1 day (30)	IR	/	NR	NR

Li 2009 [38]	53/53	SP13, SP14, SP15, ST29, ST25, ST24, ST21, RN24	MA	1 per 15 days (3)	1 per 2 days (23)	IR	/	NR	NR

Li 2007 [39]	36/36	Individualization treatment	MA	1 per 2 week (9)	1 per 2 days (63)	WC, THC, HC, FP, BM	/	NR	NR

Li 2006 [40]	30/21	ST21, ST25, RN6, ST40	MA	1 per 1 week (4)	6 per 1 week (48)	HDL	/	NR	NR

Li 2014 [41]	41/41	GB26, ST25, SP15, RN12, RN6	MA	1 per 1 week (8)	1 per 2 days (28)	/	/	NR	NR

Li 2009 [42]	72/68	Individualization treatment	MA	1 per 10 days (6)	3 per 1 week (24)	/	/	NR	NR

Liu 2008 [43]	27/28	RN13, RN12, ST36, ST25	MA	1 per 35 days (2)	1 per 1-2 day(s) (38)	/	/	NR	NR

Liu 2008 [44]	40/40	RN12, RN10, RN4, RN6, ST25, SP15, ST24, ST28	MA	1 per 8 days (3); 1 per 15 days (3)	1 per 1-2 day(s) (20)	IR	/	NR	NR

Liu 2007 [45]	32/32	ST25, RN4, RN9, ST36, ST40, SP6	MA	1 per 2 weeks (2)	1 per 2 day (14)	/	/	NR	NR

Meng 2005 [46]	48/48	ST37, ST40, SP6, SP4, ST34, ST25, BL20, BL21, BL25	MA	1 per 1 weeks (4)	1 per 1 day (30)	IR	/	NR	NR

Ruan 2009 [47]	35/35	BL25, ST25, BL21, RN12, BL27, RN4, ST37, ST40, SP9	MA	1 per 2 weeks (4)	1 per 1-2 day(s) (25)	IR, WHR	/	NR	NR

Tian 2014 [48]	22/22	RN12, LR13, ST25, RN4, SP15, ST37, ST40	MA	1 per 15 days (4)	1 per 1 day (52)	IR	/	NR	NR

Wang 2001 [49]	60/30	RN9, RN7, ST25, ST40	MA	1 per 1 month (3)	1 per 1 day (63)	/	IR	NR	NR

Wang 2006 [50]	30/30	ST37, ST40, SP6, SP4, ST34, ST25, BL20, BL21, BL25	MA	1 per 1 weeks (4)	1 per 1 day (30)	IR	/	NR	NR

Xia 2014 [51]	36/36	ST25, ST36, RN13, RN12	MA	1 per 15 days (6)	1 per 3 days (30)	/	IR	NR	NR

Xiong 2006 [52]	25/23	AShi, RN12, ST25, GB26	MA	1 per 1 weeks (8)	1 per 1 day (56)	/	/	NR	NR

Xu 2014 [53]	30/30	BL18, BL20, BL23, ST40, SP6, ST36, RN12, RN10, ST25, ST29, RN4, EX-CA1, SP9	MA	1 per 2 weeks (6)	1 per 2 days (42)	OD, BMI, WHR, IR	/	NR	NR

Yan 2007 [54]	35/33	ST25, ST40, ST36, SP6, BL21, BL20, BL25, ST28	MA	1 per 15 days (6–10)	1 per 1 day (>90)	BMI, IR	/	NR	NR

Yang 2011 [55]	30/30	RN12, ST34, RN9, RN4, ST25, SP15, LI 11, SJ6, ST44, ST40, ST37, SP6, SP9	MA	1 per 15 days (4)	6 per 1 week (30)	IR	/	NR	NR

Yao 2014 [56]	25/25	RN12, ST25, RN6, SP15, ST28, SP14, ST24, ST40, SP6	MA	1 per 10–14 days (6–8)	5 per 1 week (60)	BMI, WC, HC, OD, FP, IR	/	NR	NR

Yin 2007 [57]	27/26	RN12, RN9, RN6, RN4, ST25, SP15, ST36, SP9	MA	1 per 1 week (4)	1 per 1 day (30)	/	/	NR	NR

Zhang 2014 [58]	30/30	ST25, RN12, LI 11, SP9, ST40, LR3, ST29, RN10, RN3, SJ6	MA	1 per 1 week (8)	not clear	WC, BW, BMI, IR		NR	NR

Zhang 2007 [59]	78/47	LI 11, RN12, ST25, RN6	MA	1 per 15 days (3)	1 per 1 day (30)	IR	/	NR	NR

Zhang 2006 [60]	30/30	RN12, ST25, RN6, ST37	MA	1 per 15 days (6)	1 per 2 days (36)	/	/	NR	NR

Zhang 2008 [61]	30/30	RN12, ST25, RN6, ST37	MA	1 per 10 days (3)	1 per 2 days (45)	appetite	/	NR	NR

Nie 2007 [62]	56/70	RN12, RN4, ST25, AShi	drug (sibutramine)	1 per 20 days (6)	10 mg per 1 time, 1 time per 1 day, total 56 days	/	/	NR	headache, dry mouth, anorexia, insomnia, constipation, rapid heartbeat, mild high blood pressure

Cong 2007 [63]	32/31	RN12, RN9, ST25, BL25, ST34, SP4	drug (ZhiBiTuo)	1 per 1 week (8)	0.7 g per 1 time, 3 times per 1 day, total 8 weeks	BW, BMI, WHR	/	subcutaneous indurations (*n* = 5), red and swollen (*n* = 1)	gastrointestinal discomfort (*n* = 6)

Zhang 2010 [64]	35/34	RN12, ST25, SP15, RN6, RN4, RN3, LA14, RN9, ST40, SP6, GB30, BL37, LI 11	drug (sibutramine)	1 per 15 days (6)	10 mg per 1 time, 1 time per 1 day, total 56 days	/	/	/	*n* = 5

Li 2014 [65]	30/30	GB26, GB27, GB28, RN4, KI13, ST28, BL52	drug (metformin)	1 per 1 week (4)	not clear	IR, leptin, insulin	/	NR	NR

Zhang 2008 [66]	40/40	RN10, RN5, ST25, ST23, ST27	EA	3 per 1 week (24)	3 per 1 week (24)	WHR, BW, TC, TG	/	NR	NR

Zhu 2014 [67]	25/25	RN13, RN12, RN10, RN7, RN9, RN4, RN6, ST25, ST24, ST26, SP15, SP14, GB26, GB27, LI14, SJ13, LI4, LI 11, GB31, GB32, ST34, ST36, ST37, ST40, SP6, SP9	EA	1 per 2 weeks (4)	3 per 1 week (24)	/	/	NR	NR

Huang 2011 [68]	30/30	RN12, ST25, SP15, RN9, RN6, RN4, ST36, Ashi	EA	1 per 1 week (4)	3 per 1 week (24)	medical cost	BW, IR, BMI, WC, HC, WHR	NR	NR

Ruan 2010 [69]	30/30	ST21, ST25, ST28, Ashi, RN12, RN9, ST27, GB26, Ashi	EA	1 per 1 week (8)	3 per 1 week (24)	IR, BW, BMI, WC, HC, WHR	/	NR	NR

Guo 2009 [70]	68/65	RN12, RN10, RN6, RN4, ST40, SP15, ST24, ST36	Sham	1 per 1 week (8)	1 pre 1 week (8)	IR	/	NR	NR

Jia 2014 [71]	20/20	ST24, ST28, GB26, SP14, ST21, ST25, ST40	Sham	1 per 15 days (6)	1 per 15 days (6)	IR, BW, BMI, HC, CC, WC, HC, THC, SC	/	NR	NR

Hou 2014 [72]	40/40	RN12, ST25, RN4, RN6, ST36, ST40	Cupping	1 per 15–20 days (4)	2-3 per 1 week (8–12)	IR, WC	/	NR	NR

Pan 2009 [73]	40/40	ST25, RN12, RN4, BL25, BL20, ST24, SP15, RN9, RN7, BL21, BL26, ST26, GB26, RN11, RN6, BL23, BL28	EA	1 per 1 day (60)	3 per 1 week (20)	WHR, BMI,	/	NR	NR

Tang 2009 [74]	33/32	RN12, ST25, RN6, RN4, ST34, ST36, SP4, BL15, BL20	EA	1 per 15 days (3)	1 per 1-2 day(s) (15)	IR, BW, BMI, WC, WHR, HAMD, HAMA, PSQI	/	NR	NR

ACE: acupoint catgut embedding, MA: manual acupuncture, EA: electroacupuncture, Ct.: control, IR: improvement rate, BW: body weight, BMI: body mass index, CC: chest circumference, FP: fat percentage, THC: thigh circumference, BM: basic metabolism, HC: hip circumference, WHR: waist hip rate, BM: basal metabolism, OD: obesity degree, TC: total cholesterol, TG: triacylglycerol, SC: shank circumference, HDL: high-density lipoprotein, HAMD: Hamilton depression scale, HAMA: Hamilton anxiety scale, and PSQI: Pittsburgh sleep quality index.
